# Anomalous resonance frequency shift in liquid crystal-loaded THz metamaterials

**DOI:** 10.1515/nanoph-2022-0036

**Published:** 2022-04-21

**Authors:** Eleni Perivolari, Vassili A. Fedotov, Janusz Parka, Malgosia Kaczmarek, Vasilis Apostolopoulos

**Affiliations:** Physics and Astronomy, University of Southampton, Highfield SO17 1BJ, Southampton, UK; Laboratory for Advanced Materials Processing, Empa, Swiss Federal Laboratories for Materials Science and Technology, Feuerwerkerstrasse 39, Thun 3602, Switzerland; Optoelectronics Research Centre & Centre for Photonic Metamaterials, University of Southampton, Highfield SO17 1BJ, Southampton, UK; Institute of Applied Physics, Military University of Technology, 2 Kaliskiego Str., 00-908 Warsaw, Poland

**Keywords:** metamaterials, nematic liquid crystals, optical nonlinearity, terahertz

## Abstract

Babinet complementary patterns of a spectrally tunable metamaterial incorporating a nematic liquid crystal is normally assumed to exhibit the same tuning range. Here we show that for a hybrid, liquid crystal-loaded metamaterial, the sensitivity of its terahertz resonances to the variations of the refractive index differs substantially for the two complementary patterns. This is due to a mismatch between the alignment of the liquid crystal and the direction of the local electric field induced in the metamaterial patterns. Furthermore, and more intriguingly, our experimental data indicate that it is possible to shift the resonance of the positive metamaterial pattern beyond the limit imposed by the alignment mismatch. Our analysis suggests that the observed anomalous frequency shift results from the orientational optical nonlinearity of a nematic liquid crystal.

## 1 Introduction

Compact optical components, which can efficiently control terahertz radiation (typically associated with 0.1–10 THz band of electromagnetic spectrum), are the focus of intense research given its significant potential for applications in security screening, sensing, imaging, non-destructive evaluation and high-speed wireless communication [1], [2], [3], [4]. However, the lack of an appropriate response at these frequencies in naturally available materials renders the development of THz optical components challenging. As a result, engineering artificial materials, the so-called metamaterials [5], are becoming one of the mainstream solutions for the THz technology [6], [7], [8].

Electromagnetic metamaterials (MMs) have advanced rapidly and had a major impact in technologies spanning from RF and microwave technologies to photonics and nanophotonics [9]. The MMs concept not only has brought to life such exotic optical effects as artificial magnetism [10, 11], negative refraction [12] and cloaking [13], but also enabled dramatic enhancement of light–matter interaction leading to amplified absorption [14], asymmetric transmission [14], giant polarization rotation [15], and slow light [16, 17].

The key to the enhanced light–matter interaction in MMs is a narrowband resonant response, which can be engineered in metallic MMs at virtually any frequency within the THz band taking the advantage of very large dielectric constants of metals. Although the frequencies of MM resonances are determined by the structure of MMs and, therefore, are fixed by design, they can be tuned with the help of functional materials integrated into the fabric of MMs. Nematic liquid crystals (NLCs) were arguably the first functional media successfully exploited for active control of MMs [18, 19], offering an easy-to-implement control mechanism based on reversible refractive index change [20]. LC-loaded MMs have since become a very popular and well-researched artificial material system with many applications across the electromagnetic spectrum, and in particular, in the THz domain (see, for example, [21] and references therein).

In this letter we show that such a hybrid material system still holds some surprising and unexplored effects. In particular, we report that metallic THz MMs with Babinet complementary patterns do not necessarily exhibit the same frequency tuning range when integrated with an NLC. We also show that the difference is controlled by two opposing effects: (i) mismatch between LC alignment and the direction of local electric fields induced in the MMs, and (ii) orientational (Kerr) optical nonlinearity of NLCs. We discover that, remarkably, the integration of NLCs with metallic MMs enables the enhancement of Kerr nonlinearity to the level that it can be engaged in low-intensity experiments in the THz domain.

## 2 Results and discussion

**Figure 1:**
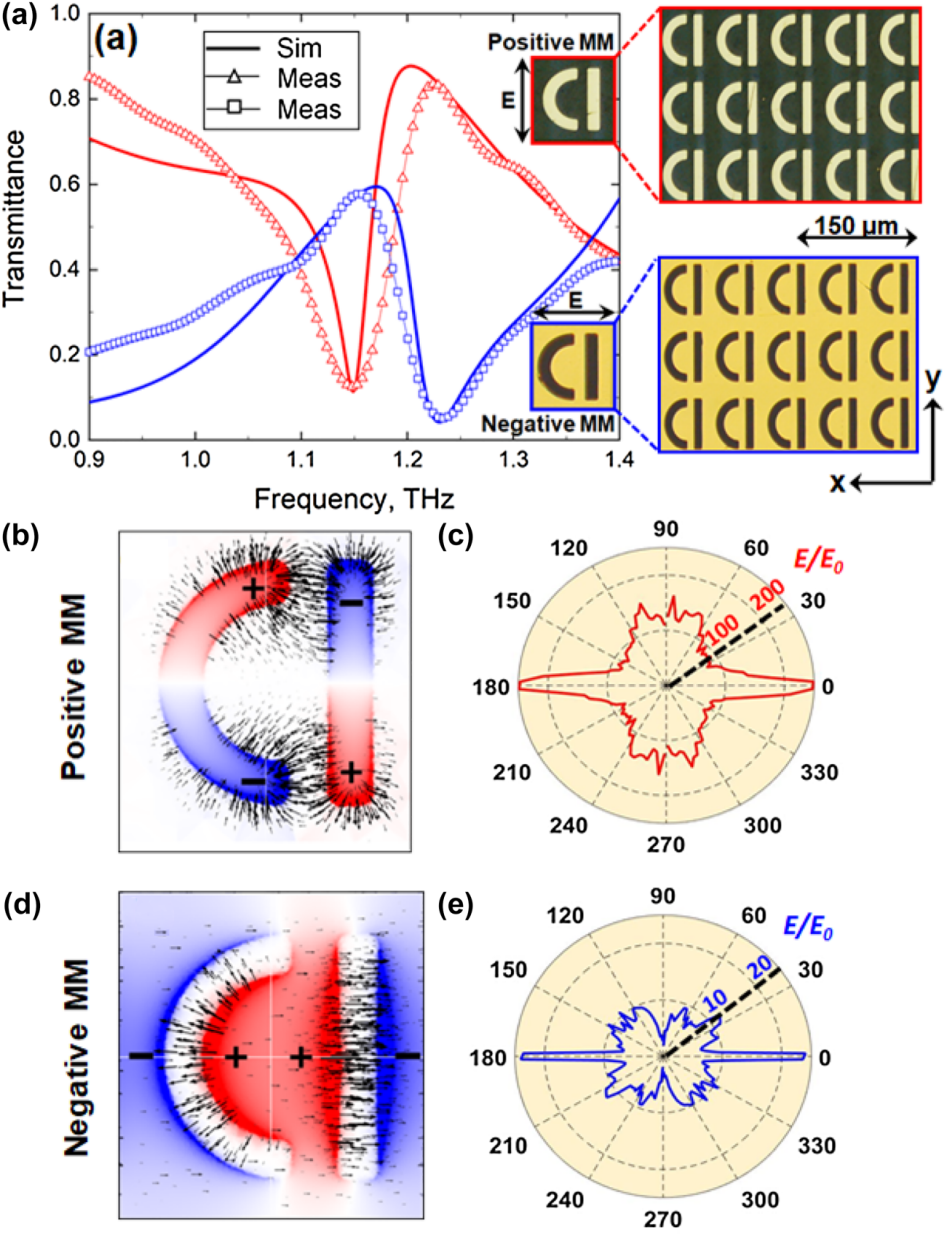
(a) Transmission spectra measured experimentally (open triangles) and simulated (thick solid lines) for pristine MMs of positive (red) and negative (blue) designs. Insets: images of the unit cells and fragments of the fabricated MMs of positive (top) and negative (bottom) designs taken with a reflection microscope. (b) Normalised distribution of the surface charge density (colours) and electric field lines (black arrows) induced in the positive MM at the resonant frequency. (c) Polar plot of in-plane directivity of the local electric field in positive MM averaged over the area of the unit cell. The radius of the plot corresponds to the magnitude of the average electric field (*E*) normalised on the incident field (*E*_0_). Fine features of the directivity diagram are numerical artefacts caused by discretisation of the MM and space above it in our model. (d) Same as (b) but for negative MM. (e) Same as (c) but for negative MM.

The design of MMs we chose for our study was based on a D-shaped metamolecule (see insets to Figure 1(a)) – a derivative of the asymmetrically-split ring resonator, which in the past had enabled the engineering of high-Q Fano resonances in MMs [16] and served as a building block for one of the first MM-based sensing platforms [22]. The two Babinet complementary patterns of the MMs featured arrays of such metamolecules formed, respectively, by metal patches (positive MM) and slits in a metal screen (negative MM), as shown in the inset to Figure 1(a). The width of the patches/slits was 10 μm, and the unit cell of the arrays had the dimensions of 70 μm×80 μm . Figure 1(a) shows the transmission spectra of both MMs simulated in COMSOL 5.5 Multiphysics for normally incident linearly polarised plane waves, assuming that the patterns were cut in a 300 nm thick gold film [23] supported by a 100 μm thick slab of fused quartz with the refractive index of 2 [24]. The spectra reveal the presence of Fano resonances for both positive and negative MMs, featuring the characteristic asymmetric profile [25, 26] with a sharp roll-off centred at around 1.17 THz and 1.22 THz, respectively. To engage Fano resonances the polarization was set parallel to the straight segments of the metamolecules for positive MM (y-polarisation), and perpendicular to the straight segments of the metamolecules for negative MM (x-polarisation), as illustrated in the insets to Figure 1(a). The transmission spectra simulated for the orthogonal polarizations were featureless, as expected.

Both resonances can be traced to the excitation of the so-called trapped modes [16]. These are weakly radiating modes, which correspond to charge oscillations induced in the two segments of the D-shaped metamolecule in anti-phase (see Figure 1(b) and (d)). The observed Fano lines result from the interference of such modes with their strongly radiating counterparts formed by in-phase oscillations of charges (see SI, Figure S1 for details). We note that the corresponding distributions of the electric field differ substantially for the resonances of positive and negative MMs. The ‘hotspots’ of the electric field appear to localise around the ends of the metal patches in the positive MM, and within the central areas of the slits in the negative MM. As a result, the extent of the in-plane divergence of the local field in the two cases is also very different. Indeed, in the positive MM the field lines are seen to fan out within the hotspots, spanning nearly all directions (see Figure 1(b)), while in the negative MM they stretch across the slits and, thus, align predominantly along the *x*-axis (see Figure 1(d)). The difference becomes more apparent when analysed in terms of the directivity of the local field averaged over the area of a unit cell (see diagrams in Figure 1(c) and (e)). Clearly, in the case of the negative MM the directivity collapses around the *x*-axis and is only perturbed by four small diagonal side lobes generated by the curved segment of the metamolecule. In the case of the positive MM, however, the directivity features four main broad lobes oriented along the x- and *y*-axes. We argue that such a directional anisotropy of the local field, which arises near the surface of the MMs, becomes an important factor in the presence of optically anisotropic media (such as NLCs and films of oriented carbon nanotubes [27, 28]) affecting differently the tuneability of Babinet complementary MMs.

**Figure 2:**
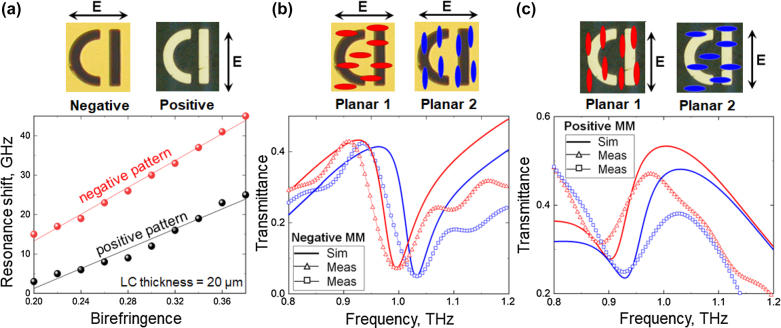
(a) Calculated maximum resonance shift that can be attained in positive (black points) and negative (red points) MMs at different values of LC birefringence. Insets: Images of MM unit cells taken with a reflection microscope. (b) Transmission spectra measured experimentally (open triangles) and simulated (thick solid lines) for negative MM loaded with NLC in planar 1 (red) and planar 2 (blue) configurations. Insets: orientations of LC molecules schematically shown for planar 1 and planar 2 configurations. (c) Same as (b) but for positive MM.

To the best of our knowledge such an effect has not been discussed in the literature. To demonstrate it first numerically, we introduced in our model a layer of an optically anisotropic dielectric, which represented an NLC in the planar state. It had the thickness of 20 μm, typical for conventional LC optical cells, and was placed on top of the MMs, fully encompassing their structure (see SI, Figure S2). The ordinary and extraordinary refractive indices of an NLC were assumed to be $n_{\text{o}}=1.574-i0.017$ and $n_{\text{e}}=1.951-i0.024$ , corresponding to the refractive indices of highly birefringent LC 1825 at 1 THz [29]. Figure 2(b) and (c) compare the simulated transmission spectra of the complementary MMs with two different planar configurations of the NLC aligned, respectively, parallel (planar 1) and perpendicular (planar 2) to the incident polarisation. Evidently, upon switching the NLC between planar 1 and planar 2 configurations, the resonance of the positive MM exhibits a spectral shift some 20 GHz (∼50%) smaller than that of the negative MM. This is the manifestation of the effect of the mismatch between the direction of the local field and LC alignment, which we referred to above. Here, the tendency of the local electric field to oscillate along two orthogonal directions in the positive MM (see diagram in Figure 1(b)) makes the supported electromagnetic mode less sensitive to the alignment of the NLC (and, correspondingly, anisotropy of its refractive index) and therefore results in a spectrally narrower tuning range.

We demonstrate the extent of the effect in Figure 2(a) by comparing the maximum frequency shifts exhibited by the resonances of the complementary MMs for different values of NLC birefringence, Δ*n* = *n*_e_ − *n*_o_. In our model Δ*n* varied in the range from 0.2 to 0.38, where the limits correspond to the birefringence of two different liquid crystals, E7 [30] and LC 1825 [29, 31] at 1 THz. The values of Δ*n* within this range were calculated based on *n*_e_ and *n*_o_ linearly interpolated between those of E7 and LC 1825. The data points in Figure 2(a) mark the difference between the resonance frequencies calculated for planar 1 and planar 2 states. As expected, the frequency shift becomes smaller for both MMs as the value of the birefringence decreases towards 0.2, yet the positive MM exhibits consistently smaller shifts than the negative MM for the entire range of Δ*n*.

The predicted difference in the tuneability of Babinet complementary MMs represents a practically important limitation. To confirm it experimentally, we fabricated the exact copies of the above MMs and characterised their spectral response at normal incidence using a conventional low-power THz-TDS setup, which featured a micro-dipole based photoconductive antenna excited with 10 mW of ultrafast (100 fs) 780 nm laser and measurement based on the Pockels effect. The patterns of the MMs were etched using UV photolithography in a 305 nm thick metal film, which had been deposited beforehand by thermal evaporation onto 1.17 mm thick fused quartz substrate. The metal film comprised a 300 nm thick layer of gold and a 5 nm thick layer of chromium added to ensured good adhesion between gold and quartz. The overall size of the fabricated samples was 16 mm × 16 mm. The terahertz beam was focussed onto the samples to a 5 mm large spot, which guaranteed the absence of beam clipping and diffraction at the edges of the samples upon transmission. The measured transmission spectra of the pristine positive and negative MMs are shown in Figure 1(a). The plot reveals a very good agreement between the experimental and modelled data confirming high quality of the fabricated MM samples. The faint high-frequency modulations of the experimental curves are artefacts, which resulted from the use of a time window function in the frequency-domain analysis of the transmitted THz pulses. Although impossible to avoid, those artefacts were minimised by carefully adjusting the shape of the window function.

Each MM was functionalised with LC 1825 via the planar cell arrangement, where a 20 μm thick layer of the NLC was sandwiched between the MM and a pristine slab of fused quartz (1.17 mm thick), as schematically shown in Figure S2. The surface of the MM and the surface of the quartz slab facing the NLC were both coated with a thin film (∼30 nm) of uniformly rubbed polyimide (PI-2525 from HD MicroSystems). Such a film acted as an alignment layer, which promoted the orientation of LC molecules near its surface in the direction of rubbing. The planar alignment of LC 1825 in the bulk was ensured by matching the directions of rubbing at the opposite sides of the resulting optical cell. To minimise fabrication inconsistencies here, the two variants of the planar alignment, namely planar 1 and planar 2, were produced simultaneously in different parts of the same cell.

The transmission spectra of LC-loaded MMs, as measured with our THz-TDS setup, are presented in Figure 2(b) and (c). Clearly, the locations of the resonance exhibited by the negative MM in the presence of differently aligned NLC (i.e., in planar 1 and planar 2 configurations) agree very well with the predictions of our model, with the spectral separation reaching the expected 45 GHz. In the case of the positive MM, however, a good spectral overlap between the experimental and modelled data is seen only for the NLC in planar 2 configuration. Surprisingly, switching the NLC to planar 1 configuration red-shifts the measured transmission spectrum of the positive MM by about 20 GHz further than what seems to be allowed from the simulation. As a result, the frequency tuning range obtained experimentally for the positive MM widens to 43 GHz, which practically negates the large difference in the tuneability of Babinet complementary MMs predicted by our simulations. Such an unexpected discrepancy between the simulation and experiment, whereby the complementary forms of the MM end up being equally sensitive to the anisotropy of NLC refractive index (even in the case of the largest available anisotropy, Δ*n* = 0.38), calls for a careful examination of the anomalous behaviour. Below we provide such an analysis, which forms the core part of the study reported in this paper.

Given that a large frequency mismatch between the measured and simulated spectra was observed only in one case, we ruled out an uncertainty in specifying the refractive index of LC 1825 as a possible explanation of the discrepancy. We reason that the larger red-shift of the MM spectrum could have resulted only from a better match between the direction of the local electric field and the orientation of LC molecules, since that would effectively increase the refractive index of the NLC perceived by the resonant electromagnetic mode of the metamolecules. To this end we note that the directional spread of the local field in the positive MM is largest around the *y*-axis (see diagram in Figure 1(b)) and, hence, matching the spread with local distortions of the NLC will have the biggest impact for planar 1 alignment – exactly where the outcomes of our simulations and experiment disagree the most. The above considerations allow us to readily exclude the local heating of NLC within the hotspots and, as a result, its transition to an isotropic phase as a potential mechanism of the effect. Indeed, in the isotropic phase the orientation of LC molecules is random and, therefore, the refractive index perceived by the resonant electromagnetic mode cannot be maximised for all directions of its local electric field. We further confirmed this with rigorous modelling, which shows that the spectral shift in the case of local heating would in fact occur in the opposite direction (see SI, Figure S4 for details).

**Figure 3:**
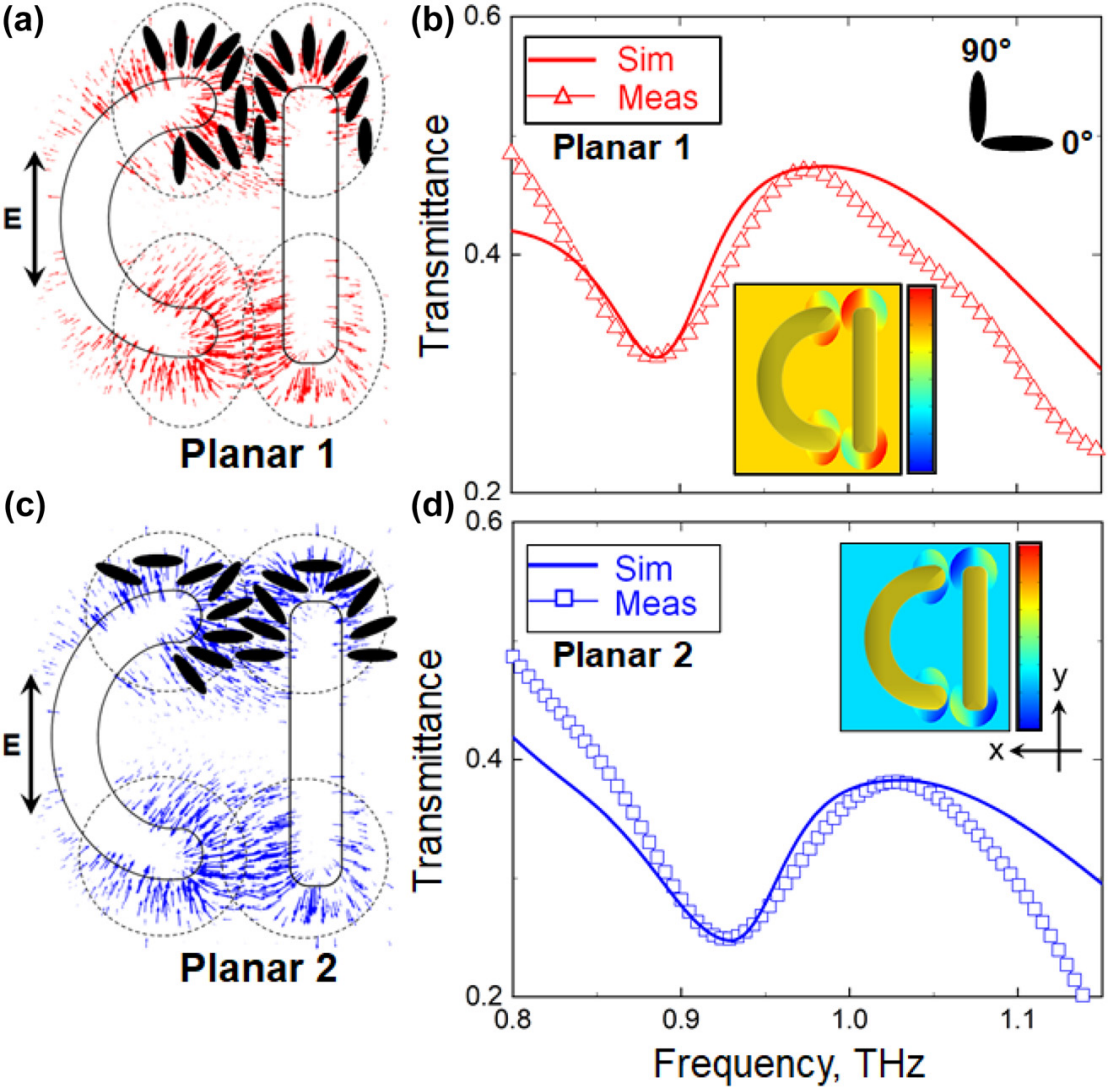
(a) Orientational optical nonlinearity of NLC in planar 1 configuration engaged with positive MM. Local field-induced distortions of the initial alignment assumed within the hotspots are shown schematically via the orientation of LC molecules. (b) Transmission spectra measured experimentally (open triangles) and simulated (thick solid lines) for positive MM taking into account the effect of orientation nonlinearity of NLC in planar 1 configuration. Inset: spatial distribution of LC alignment assumed in the simulations. Scale bar ranges from −45° (blue) to 135° (red). 0° and 90° correspond to LC molecules being parallel to *x*- and *y*-axes, respectively. (c) Same as (a) but for initial planar 2 alignment of NLC. (d) Same as (d) but for initial planar 2 alignment of NLC.

To demonstrate the plausibility of our hypothesis we re-modelled the response of the positive MM assuming that the NLC aligned itself in the direction of the local field within the hotspots (where the divergence of the field is strongest), and such distortions were confined to a distance of 5 µm away from the edges of the metamolecules and 1 µm above their plane (see SI, Figure S3). We further assumed that changes in LC alignment there were limited to [−45°, +45°] range of angles, which corresponded to the angular spread of the local field we noted above. For the directions outside the spread the orientation of LC molecules was gradually restored to the background alignment (i.e., planar 1 configuration), as schematically illustrated in Figure 3(a) and (c). The transmission spectrum of the positive MM calculated using our modified model is presented in Figure 3(b), where it is compared with the spectrum measured experimentally. Evidently, this time the predicted and the actual locations of the MM resonance coincide. Moreover, a very good agreement between the simulated and experimental data is now also seen in terms of the amplitude of the MM transmissionl. As in the case of a pristine MM, the faint high-frequency modulation of the spectrum visible above 1 THz is an artefact, which resulted from the use of a time window function in the frequency-domain analysis of the transmitted THz pulses.

As a further test of our hypothesis, we modified the model of the positive MM with the NLC featuring planar 2 alignment. Localised distortions of the alignment were introduced in the model in the same way as in the planar 1 case except that the re-orientation of LC molecules was traced relative to the *x*-axis (see Figure 3(d)). What we found was that assuming re-alignment of the NLC within the hotspots even in the planar 2 case (where the theory and experiment had already agreed qualitatively) dramatically improved the accuracy of our model and enabled us to achieve nearly perfect quantitative agreement between the calculated and measured spectra (see Figure 3(d)). For completeness, in Figure S5 of the Supporting Information we present the transmission spectra of the positive MM simulated for planar 1 and planar 2 cases assuming LC re-orientation within, respectively, smaller and larger range of angles. Evidently, local re-orientation limited to angles [−30°, +30°] and [−90°, +90°] results in poor agreement with the experiment.

In principle, the admitted local re-orientation of the NLC could signify alignment defects, which might have formed at the edges of the metamolecules. We examined the samples under a polarised microscope before and after the measurements but found no deviations from uniform planar alignment of the NLC. Given that the appearance of the anomalous spectral shift was a repeatable effect, while the re-alignment of the NLC must occur within the hotspots in simultaneously all metamolecules upon illumination of the sample, it could not be a spontaneous process. We, therefore, conclude that it must have been induced by the electric fields of the metamolecules and so the anomalous frequency shift observed experimentally for the resonance of the positive MM was a manifestation of Kerr (orientational) optical nonlinearity of the NLC [32].

To the best of our knowledge, Kerr nonlinearity of LCs has not been reported for the intensities accessible with conventional commercial THz-TDS setups (typically built around mW lasers pumping photoconductive emitters) and we argue that the following factors could have enabled the observation of the effect in our experiments. We note, firstly, that the re-orientation of the NLC in the configuration described here is equivalent to in-plane electrical switching, which would occur without a threshold for the initial misalignment of the NLC of less than 45°, as follows from [33]. We further note that since the local electric field has an out-of-plane component, the re-orientation of LC molecules within the hotspots should also occur out of plane and, hence, our model underestimates the strength of the NLC nonlinear response. Secondly, the E-field enhancement in our structures is dominated by two combined effects. One effect is the amplification of THz fields by the MMs via their high-Q resonance. The other effect is the concentration of electric fields near sharp geometric features of the metamolecules, the so-called ‘lightning rod’ effect [34, 35]. The first (resonant) effect is relevant to both the negative and positive MMs and enables the generation of local fields one order of magnitude stronger than the incident field (as evident from diagrams in Figure 1(c) and (e)). The second (geometrical) effect ensures further ten-fold enhancement only in the positive MM, where sharp geometric features of the metamolecules and localisations of the resonantly amplified fields (i.e., hotspots) coincide. As a result, the overall field enhancement attainable in the positive MM is likely to exceed two orders of magnitude (see diagram in Figure 1(c)). We, therefore, argue that the combination of high E-field enhancement and threshold-less nature of the LC re-orientation make it not unreasonable to expect that the local field induced in the positive MM would enable optical switching of the NLC even at the intensity levels characteristic of our THz-TDS setup.

## 3 Conclusions

In conclusion, we demonstrate that Babinet complementary patterns of a THz metallic MM do not exhibit the same frequency tuning range when hybridized with an NLC. The results of our study suggest that the difference results from a mismatch between the alignment of the NLC and the direction of the local electric field induced in the patterns. More intriguingly, our experimental observations indicate that it is possible to shift the resonance of the positive MM pattern beyond the limit imposed by the alignment mismatch and to significantly increase its tuning range. Our analysis suggests that the observed anomalous frequency shift results from the orientational optical nonlinearity of the NLC enhanced via integration with the metallic MM. We envisage that our findings can directly lead to the increase of the efficacy of THz modulators and other active optical components exploiting the enhanced nonlinear light–matter interactions in LC-MM hybrid structures. Most importantly, we show that nonlinear effects can be engaged with a low power photoconductive antenna-based THz spectrometer, when combined with resonant amplification and sub-wavelength concentration of THz fields facilitated by MMs. This demonstration enables future studies of nonlinear effects in the THz range, which up to now, where exclusively reserved for high-power THz spectrometers based on complex and often cumbersome amplified laser systems.

## Supplementary Material

Supplementary Material Details
