# Supplementary material for: Anomalous resonance frequency shift in liquid crystal-loaded THz metamaterials
Source: Nanophotonics. 2022 Apr 21;11(10):2341–8. doi: 10.1515/nanoph-2022-0036 (PMC11636508; doi:10.1515/nanoph-2022-0036)
Supplement: Supplementary file 1 — Supplementary Material Details [file j_nanoph-2022-0036_suppl.docx]

**Supporting Information**

# Anomalous Resonance Frequency Shift in Liquid Crystal-Loaded THz Metamaterials

Eleni Perivolari^1,2*^, Vassili A. Fedotov^3^, Janusz Parka^4^, Malgosia Kaczmarek^1^, and Vasilis Apostolopoulos^1^

1. *Physics and Astronomy, University of Southampton, Highfield SO17 1BJ, UK*
2. *Laboratory for Advanced Materials Processing, Empa, Swiss Federal Laboratories for Materials Science and Technology, Feuerwerkerstrasse 39, Thun 3602, Switzerland*
3. *Optoelectronics Research Centre & Centre for Photonic Metamaterials, University of Southampton, Highfield SO17 1BJ, UK*
4. *Institute of Applied Physics, Military University of Technology, 2 Kaliskiego Str., 00-908 Warsaw, Poland*
5. **The origin of the Fano resonances**


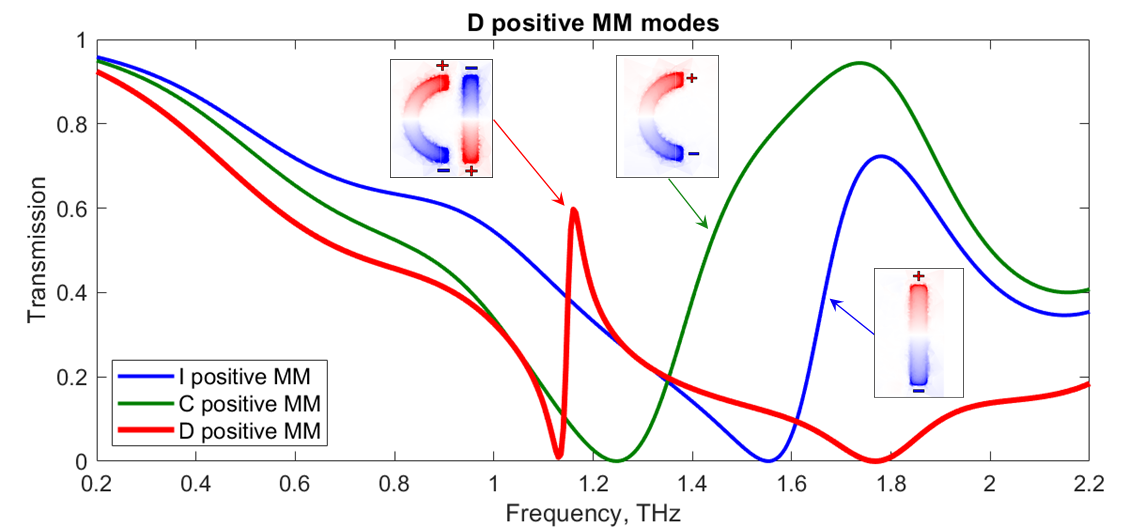


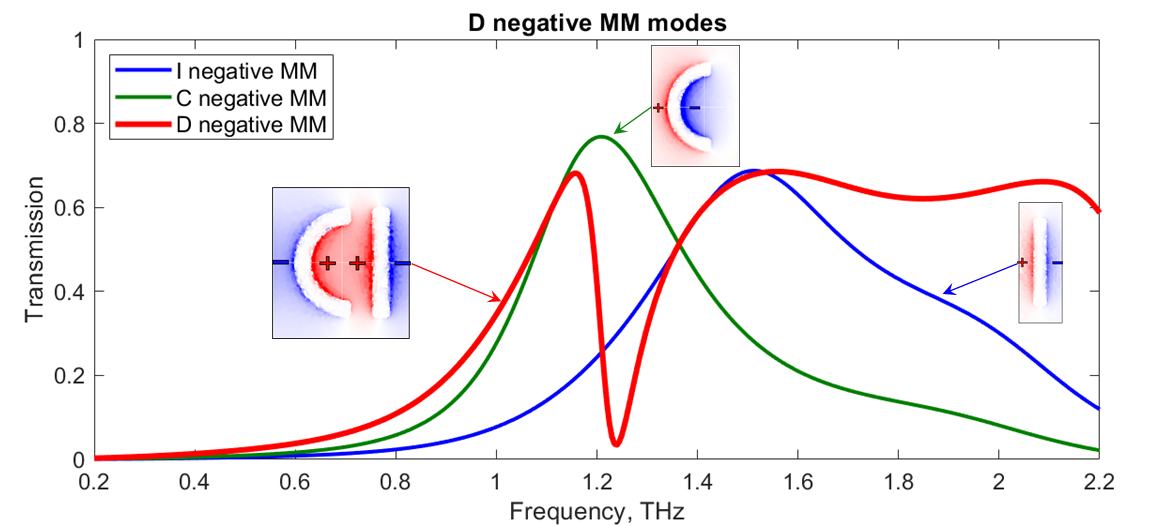


**Figure S1.** Schematic Transmission spectra simulated for pristine MMs of positive (top) and negative (bottom) D-, I- and C-shaped MM designs. Insets: Normalised distribution of the surface charge density induced in the MMs at the resonant frequency.

The origin of the Fano resonances arises from the constructive and destructive interference of a narrow discrete resonance with a broad spectral line from the partition of D-shaped MM structural elements that involve ‘I-shaped’ and ‘C-shaped’ MM patches and slits (positive and negative MM designs respectively). The broad line corresponds to a symmetric current mode of the metamolecule, which is an electric dipole. The narrow line corresponds to an anti-symmetric current mode, which is a magnetic dipole/electric quadrupole. Thus, the Fano line here results from the interference between the dipole and magnetic dipole/electric quadrupole modes (as shown in Figure S1), where we demonstrate the strong mode coupling between the electric dipole and magnetic dipole/electric quadrupole moments. At the frequencies of 1.67 and 1.44 THz I- and C-shaped elements of positive MMs exhibit their fundamental resonances, where the wavelength of excitations is twice the length of MM elements. The fundamental resonances of I- and C-shaped elements of the negative pattern appear at 1.51 THz and 1.21 THz, respectively. Our calculations show that in the case of D-shaped MM an antisymmetric current mode (quadrupole mode) can dominate the usual symmetric one (dipole mode). This is mainly because anti-symmetric current mode produces very weak scattered electromagnetic fields, which dramatically reduces radiation losses. Thus, by combining the symmetric dipole modes of structures ‘I’ and ‘C’, producing the ‘D-shaped’ MM, the Fano resonance combines the transmission spectra of their fundamental ones yielding to a sharper and narrower resonance. With that definite resonance position, we were able to track, in a straightforward way, even the smallest shift in the electromagnetic spectrum.

1. **LC-loaded MM hybrid cell**


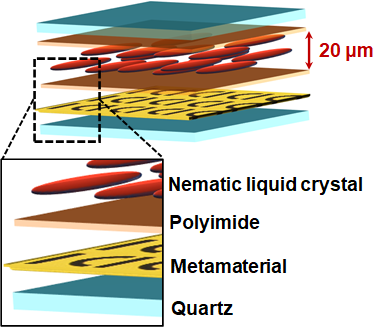


**Figure S2.** Schematic of LC-loaded MM hybrid cell used in the experiment. MM was functionalised with NLC 1825 via the planar cell arrangement, where a 20 μm thick layer of the NLC was sandwiched between the MM and a pristine slab of fused quartz (1.17 mm thick). The surface of the MM and the surface of the quartz slab facing the NLC were both coated with a thin film (~30 nm) of uniformly rubbed polyimide (PI-2525 from HD MicroSystems). Such a film acted as an alignment layer, which promoted the orientation of LC molecules near its surface in the direction of rubbing.

1. **Modelling the change of LC birefringence induced by local fields within ‘hotspots’**

The hotspots are localised at the ends of the metal patches in the positive MM, as schematically shown in Figure S3. The layer of the NLC covering the MM was assumed to be aligned along the y-axis, except in the hotspots, where maximum localisation of electric field occurs. Birefringent materials such as the NLC experience double refraction, whereby light is split, depending on the incident polarization, into two, i.e. ordinary ($n_{o}$) and extraordinary ($n_{e}$) polarizations. The extraordinary one is parallel to the LC director, $\vec{n}$, of NLC molecules, pointing along the y-axis, which is the resonant polarization. This leaves the ordinary to be parallel to the x-axis direction.


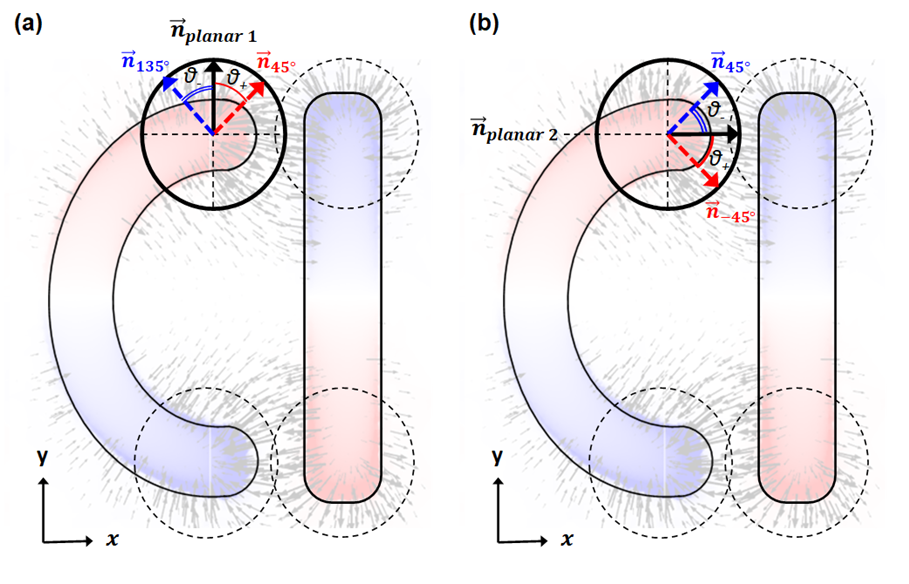


**Figure S3.** Schematic representation of modelling the anisotropy induced to the NLC molecules in **(a)** planar 1 and **(b)** planar 2 configurations due to the charge density distribution at the surface of positive MM patterns.

In order to model such optical behaviour, a diagonal optical anisotropy must be introduced in our calculations as well as a full tensor relative permittivity approach. Here, tensor refers to a 3-by-3 matrix that has both diagonal ($\epsilon_{xx},\epsilon_{yy},\epsilon_{zz}$) and off-diagonal ($\epsilon_{xy},\epsilon_{xz},\epsilon_{yx},\epsilon_{yz},\epsilon_{zx},\epsilon_{zy}$) terms, as shown below.

$$\epsilon=\left[ \begin{matrix} \epsilon_{xx} & \epsilon_{xy} & \epsilon_{xz} \\ \epsilon_{yx} & \epsilon_{yy} & \epsilon_{yz} \\ \epsilon_{zx} & \epsilon_{zy} & \epsilon_{zz} \end{matrix} \right]$$

NLCs belong to the uniaxial crystals’ category, where only the diagonal elements of permittivity tensor are nonzero. This means that $\epsilon_{xx}=\epsilon_{zz}={n_{o}}^{2} - {k_{o}}^{2}+2in_{o}k_{o} and \epsilon_{yy}={n_{e}}^{2} - {k_{e}}^{2}+2in_{e}k_{e}$. The material used in this study, LC1825, was fully investigated by Chodorow, U., et al. [27], who provided the following characteristics which we used in the simulations:

| LC1825 | 1.5 THz |
| --- | --- |
| $n_{o}$ | 1.574 |
| $n_{e}$ | 1.951 |
| $k_{o}$ | 7 [$1/{cm}$] |
| $k_{e}$ | 12 [$1/{cm}$] |
| $\Delta n$ | 0.38 |

So, for planar 1 configuration we introduced the following relative permittivity tensor with diagonal elements.

$$\epsilon=\left[ \begin{matrix} \epsilon_{xx} & 0 & 0 \\ 0 & \epsilon_{yy} & 0 \\ 0 & 0 & \epsilon_{zz} \end{matrix} \right]$$

To model the spatial variation of optical anisotropy as specified in figure 3 (a, c), we introduced an off-diagonal transverse anisotropy in the XY plane. When the $\vec{n}$ of NLC lies and rotates in the XY plane, making an angle of $\vartheta$ with the x-axis (see Figure S3), the diagonal components ($\epsilon_{xx},\epsilon_{yy},\epsilon_{zz}$) and off-diagonal components ($\epsilon_{xy},\epsilon_{yx}$) are nonzero.

$$\epsilon=\left[ \begin{matrix} \epsilon_{xx} & \epsilon_{xy} & 0 \\ \epsilon_{yx} & \epsilon_{yy} & 0 \\ 0 & 0 & \epsilon_{zz} \end{matrix} \right]$$

In that case, $\epsilon_{yy}$ is governed by the extraordinary refractive index, because $\vec{n}$ lies along the principal y-axis, while $\epsilon_{xx}$ and $\epsilon_{zz}$ are governed by the ordinary refractive index. The off-diagonal elements $\epsilon_{xy},\epsilon_{yx}$ are derived from the multiplication of the matrices as stated below.

$$\epsilon=\left[ \begin{matrix} \cos(\vartheta) & -\sin(\vartheta) & 0 \\ \sin(\vartheta) & \cos(\vartheta) & 0 \\ 0 & 0 & 1 \end{matrix} \right]\left[ \begin{matrix} \epsilon_{xx} & 0 & 0 \\ 0 & \epsilon_{yy} & 0 \\ 0 & 0 & \epsilon_{zz} \end{matrix} \right]\left[ \begin{matrix} \cos(\vartheta) & \sin(\vartheta) & 0 \\ -\sin(\vartheta) & \cos(\vartheta) & 0 \\ 0 & 0 & 1 \end{matrix} \right]=\left[ \begin{matrix} (\epsilon_{xx}){cos}^{2}(\vartheta)+(\epsilon_{yy}){sin}^{2}(\vartheta) & (\epsilon_{xx})sin(\vartheta)cos(\vartheta)-(\epsilon_{yy})sin(\vartheta)cos(\vartheta) & 0 \\ (\epsilon_{xx})sin(\vartheta)cos(\vartheta)-(\epsilon_{yy})sin(\vartheta)cos(\vartheta) & (\epsilon_{yy}){cos}^{2}(\vartheta)+(\epsilon_{xx}){sin}^{2}(\vartheta) & 0 \\ 0 & 0 & \epsilon_{zz} \end{matrix} \right]$$

To model the distortions of LC alignment within the hotspots, we isolated the areas around the edges of MM and built the tensor for each area individually. For convenience, we set a new origin ($x_{0},y_{0}$) in each area as a point of reference to rotate the molecules’ alignment, and hence the optical anisotropy, with respect to spatial coordinates.

Rotation angle $\vartheta$, will then be:

$$\hat{\vartheta}={\hat{\varphi}-tan}^{-1} \left[ \left( x-x_{0} \right)/\left( y-y_{0} \right) \right],$$

The resulting rotating angle $\vartheta$ varies from 135 to 45 degrees for planar 1 configuration (see Fig. 3b) and from 45 to $-45$ degrees for planar 2 configuration (see Fig. 3d), showing a realistic idea of what we expected, as schematically shown in Figure S3 (a, b) respectively. Angle $\varphi$, defines whether the variation axis symmetry exists at 90° along $n_{planar 1}$ or at 0° along $n_{planar 2}$.

1. **Heating of MM metal elements in the hotspots (HS)**

The heating of LCs within hotspots could well serve as an alternative mechanism of LC nonlinear response and, therefore, might also affect the spectral shift of the metamaterial resonance. The temperature effect, in particular in the area of the hotspots, was considered. Firstly, LC 1825 has high phase transition temperature, with the nematic to isotropic clearing point as high as 133^o^C. Our experiments were carried out at room temperature and this LC has negligible absorption in the THz range (the THz source had low power). Hence, direct heating of the LC through its absorption was assumed to be negligible. Secondly, heating of MM metal elements in the hotspots could, indeed, be another source of temperature variation. However, our modelling shows that in the case of local heating, when the temperature increase is enough to induce the isotropic phase within the hotspots, the spectral shift would in fact occur in the opposite direction (see Figure S4).


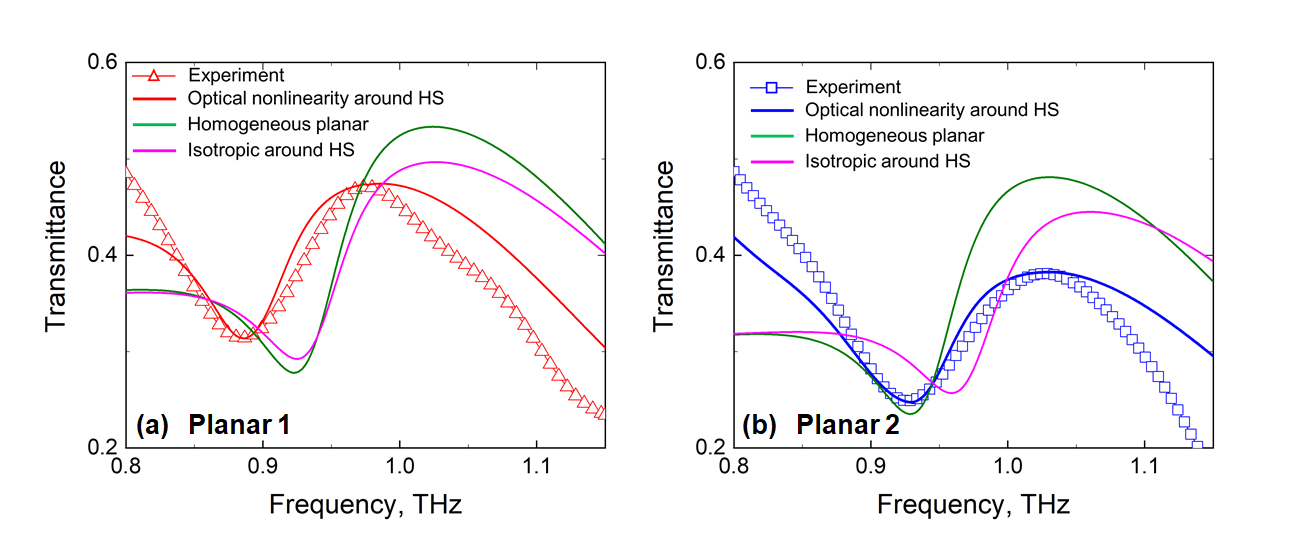


**Figure S4. (a)** Transmission spectra measured experimentally (open triangles) and simulated (thick solid lines) for positive MM including also the initial homogeneous planar 1 LC orientation (green line), the orientational optical nonlinearity caused by the enhanced near-field around MM hotspots (HS) (red line) and the isotropic LC alignment around MM HS caused by the hypothetical temperature increment (magenta line). **(b)** Same as (a), but for the initial planar 2 alignment of LC molecules.

1. **Varying the extent of LC re-alignment within the hotspots**

We simulated different angular ranges of possible LC re-alignment for both planar 1 and planar 2 configurations in an attempt to match the experimental data. Evidently, re-orientation of LC molecules within ± 45° (90°±45° and 0°±45°, respectively) found to produce best possible match to the experiment (see Figure S5). A re-orientation of ± 90° opposes the initial strong surface anchoring energy due to rubbing and, hence, seems unlikely to occur in our experiments. Indeed, as it is shown in Figure S5, re-orientation of ± 90° yields larger transmittance than observed in the experiment. On the other hand, a smaller re-orientation range of ± 30° shows a weaker response. We thus conclude that the best fit is obtained for the re-orientation of LC molecules occurring within the hotspots in the range of ± 45°.


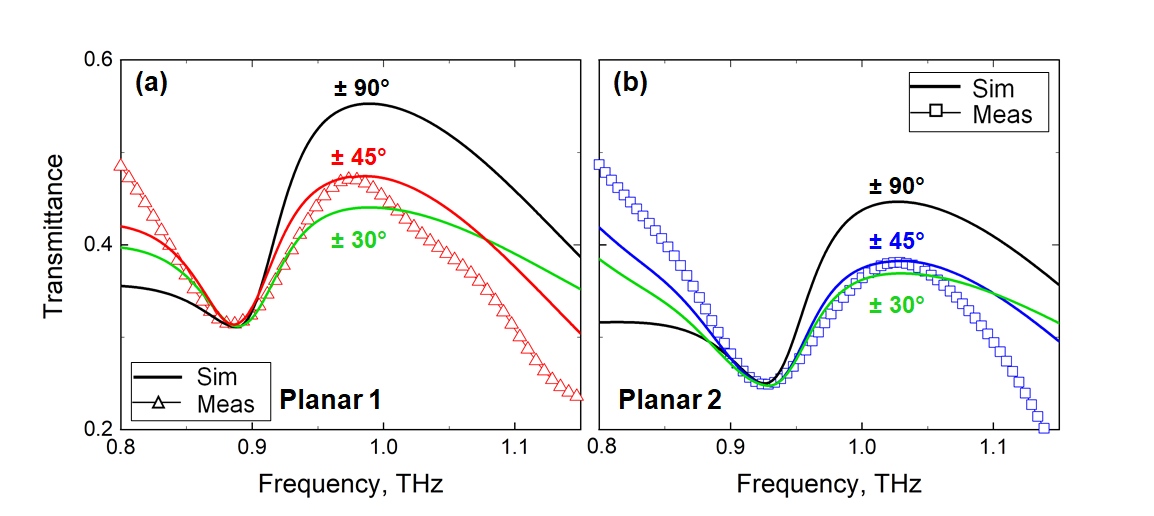


**Figure S5.** Transmission spectra of measured experimentally (open triangles) and simulated (thick solid lines) for LC-loaded positive MM in **(a)** planar 1 and **(b)** planar 2 configurations.
